# A Novel Real-Time Reference Key Frame Scan Matching Method

**DOI:** 10.3390/s17051060

**Published:** 2017-05-07

**Authors:** Haytham Mohamed, Adel Moussa, Mohamed Elhabiby, Naser El-Sheimy, Abu Sesay

**Affiliations:** 1 Department of Geomatics Engineering, University of Calgary, Calgary, AB T2N 1N4, Canada; amelsaye@ucalgary.ca (A.M.); elsheimy@ucalgary.ca (N.E.-S.); 2 Department of Electrical and Computer Engineering, Port-Said University, Port-Said 42526, Egypt; 3 Public Works Department, Ain Shams University, Cairo 11566, Egypt; mmelhabi@ucalgary.ca; 4 Department of Electrical and Computer Engineering, University of Calgary, Calgary, AB T2N 1N4, Canada; sesay@ucalgary.ca

**Keywords:** scan matching, SLAM, laser range finder, point registration, least squares, line tracking, PCA, ICP, UAV, key frame

## Abstract

Unmanned aerial vehicles represent an effective technology for indoor search and rescue operations. Typically, most indoor missions’ environments would be unknown, unstructured, and/or dynamic. Navigation of UAVs in such environments is addressed by simultaneous localization and mapping approach using either local or global approaches. Both approaches suffer from accumulated errors and high processing time due to the iterative nature of the scan matching method. Moreover, point-to-point scan matching is prone to outlier association processes. This paper proposes a low-cost novel method for 2D real-time scan matching based on a reference key frame (RKF). RKF is a hybrid scan matching technique comprised of feature-to-feature and point-to-point approaches. This algorithm aims at mitigating errors accumulation using the key frame technique, which is inspired from video streaming broadcast process. The algorithm depends on the iterative closest point algorithm during the lack of linear features which is typically exhibited in unstructured environments. The algorithm switches back to the RKF once linear features are detected. To validate and evaluate the algorithm, the mapping performance and time consumption are compared with various algorithms in static and dynamic environments. The performance of the algorithm exhibits promising navigational, mapping results and very short computational time, that indicates the potential use of the new algorithm with real-time systems.

## 1. Introduction

In recent decades, unmanned aerial vehicles (UAVs) have become an active area of research and development because of their ability to extend human capability that allows them to execute dangerous tasks safely, saving time, and, more importantly, saving lives. Furthermore, their increasing relevance stems from the potential diversity of their use both outdoors and indoors. Beyond mere observation and surveillance tasks, UAVs are increasingly being used as part of search and rescue operations, providing real-time mapping of the environment, locating victims and hard-hit areas after a natural disaster. In conjunction with UAVs, recent advances in hardware and software have made it possible to conduct accurate mapping without using costly, high-end data acquisition systems. Low-cost laser scanners and navigation systems provide accurate mapping if properly integrated at the hardware and software level. As such, UAVs have emerged as an aerial mapping platform providing additional economic and practical advantages, and thus increasing the reliability and accuracy of mapping applications [1]. To do this effectively, however, the automation of UAVs requires improvements of the vehicles’ navigation parameters. Vehicle autonomy is commonly defined as the capability of a vehicle to make decisions using sensor observations without human intervention. Hence, vehicle localization is the main step in achieving autonomy and remains a fundamental challenge for operation in unknown indoor environments. 

In addition to the localization problem, unmanned vehicles encounter several navigation hurdles, including, but not limited to: (a) the necessity of building a map of the environment during flight so the map is built from different positions. Afterward, these partial representations require assembly in order to construct a coherent map, in turn requiring the unmanned vehicle to know its position which is a challenge in indoor environments, and (b) the problem of feature extraction in sensory processing. Specifically, correct data association, used to estimate the transformation between two consecutive frames, suffers from contaminated raw measurements caused by random noise.

Therefore, navigation of a vehicle in an unknown, indoor environment is addressed by simultaneous localization and mapping method (SLAM) methods [2]. This method is a system for constructing a map of an unknown environment while concurrently estimating the position of a moving object within that environment. Typically, SLAM approaches that utilize laser range finders depend on the scan matching method of the successive scans. Scan matching is a method that has been adopted to estimate the relative transformation parameters between consecutive scans. It is performed by matching the current scan frame with either the previous scan frame or the partially built map, namely local and global scan matching, respectively [3,4]. Both approaches suffer from accumulated errors and high time consumption [5]. Many research works and implementations such as [6,7] have been published in local and global scan matching respectively.

Current approaches such as Iterative Closest Point (ICP) [8], Iterative Matching Range Point (IMRP) [9], Iterative Dual Correspondence (IDC) [9], Polar Scan Matching (PSM) [3], and Iterative Closest Line (ICL) [10] handle the scan matching problem in an iterative fashion, significantly impacting the amount of time spent on the task. Moreover, the solution convergence is not guaranteed, especially in cases of aggressive manoeuvers or rapid movement due to harsh assignment of correct correspondences [11]. Furthermore, these approaches suffer from error accumulation over time as well. Typically, a loop closure approach is used to mitigate this error [12].

Even though the SLAM method addresses the problem of navigating in unknown environments, it encounters essential mapping challenges, for example, in unstructured, dynamic, or large scale environments [13]. Indeed, the complexity increases when working in unknown unstructured dynamic environments. The environment is deemed to be dynamic when it contains objects, other than the unmanned vehicle that move and change their position over time even periodically or in random motions [14].

In dynamic environments, mapping and localization are a challenging task because the unmanned vehicles must be able to cope with the changing position of the moving objects, and furthermore, eliminate their impacts during modeling the environment [15]. People are typical examples of moving objects. There are different approaches dealing with dynamic environments [14]. First, the parameters of the moving objects are added in the state vector, then their locations are estimated. However, this approach will insert burden to the computational process. Second, much simpler approach, the dynamic entities are excluded as they are treated as noise. Third, probabilistic approaches, such as occupancy grid, are utilized due to their capability to deal with sensor noise. However, their drawback arises from the time taken to reveal that a cell is not occupied. Memory space is also a fundamental aspect in the occupancy grid, this space is proportional to the grid cell dimension [16].

Moreover, the probabilistic occupancy grid assumes that each individual cell of the grid is independent from its neighbors [17]. Each occupancy cell probability is based on information from previous observations, as well as new observations from the sensors. Unfortunately, all sensors are influenced by several sources of noise, thereby affecting their measurement accuracy. Thus, Bayesian reasoning is employed for estimating the posterior probability of the cell in order to accommodate such sensor noise [18].

Unstructured environments are characterized by no specific pattern for the environments [19]. Extracting features is exacerbated when the environment is unstructured, and often feature-to-feature methods fail to treat such environments [20].

This paper is organized as follows: Section 2 describes the origin of the idea and the overview structure of the proposed algorithm. Section 3 explains the methodology used. The experimental results are presented and discussed in Section 4. Finally, the conclusions are given in Section 5.

## 2. Overview of the Proposed Algorithm

**Figure 1:**
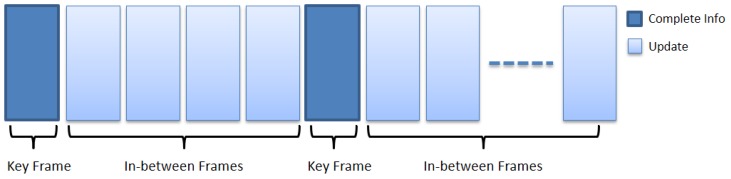
The structure of the key frame concept.

The key frame concept, in a video streaming broadcast process, is based on reducing the bandwidth load and extracting valid information from the video [21,22]. This is achieved by sending keyframes in different intervals, that provide a full summary of the video content while the in-between frames contain the update pixels only as shown in Figure 1.

From the same perspective, the proposed algorithm extracts lines from the laser range finder point cloud because line matching is more robust than point matching, and furthermore, lines are robust to the disruption of the moving objects in the dynamic environments [23]. Therefore, two non-parallel lines are chosen from the extracted lines to be the mapping reference lines as described in Section 3.2. These two reference lines play the role of the initial reference key frame of the map. Thereafter, the two reference lines are successively matched in the in-between scan frames in order to compute the transformation between the current scan frame with the reference key frame. Due to the vehicle’s movement, the existing reference lines of the current key frame might not be detectable all the time. Therefore, another reference key frame is created with two new reference lines which marks the beginning of new transformation. The transformation parameters between the old and new reference lines are computed every transition. Consequently, the last transformation parameters are computed relative to the first reference key frame. As a result, the proposed algorithm does not depend on the transformation history, and further, the effect of rotation error at any epoch between two successive frames will disappear in the upcoming transformation. Therefore, the proposed algorithm mitigates the accumulated errors by using reference lines method. Finally, the ICP algorithm is applied as a fine tuning process for the global scan matching. In case of lines outage period, due to navigating in corridor or unstructured environment, the proposed algorithm will alternate to the ICP algorithm alone. The ICP algorithm is performed for local and global scan matching as well for map consistency. Implementation of the ICP algorithm, during the lines outage period, also decreases the sensitivity of the proposed algorithm to the thresholds values because accepting reference lines depends on thresholds, discussed later, and tuning the thresholds is an important process but deploying the ICP algorithm reduces the thresholds tuning process.

**Figure 2:**
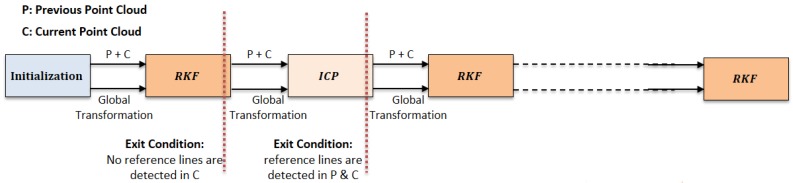
Alternating structure of the proposed algorithm between the RKF and ICP methods.

Figure 2 depicts the alternating structure between the RKF and ICP methods. As long as the proposed algorithm can choose two reference lines from the detected lines of the current scan frame, the RKF method is implemented. Otherwise, the ICP method is performed. If reference lines are detected once more in both previous and current scan frame, the proposed algorithm alternate back to the RKF method.

**Figure 3:**
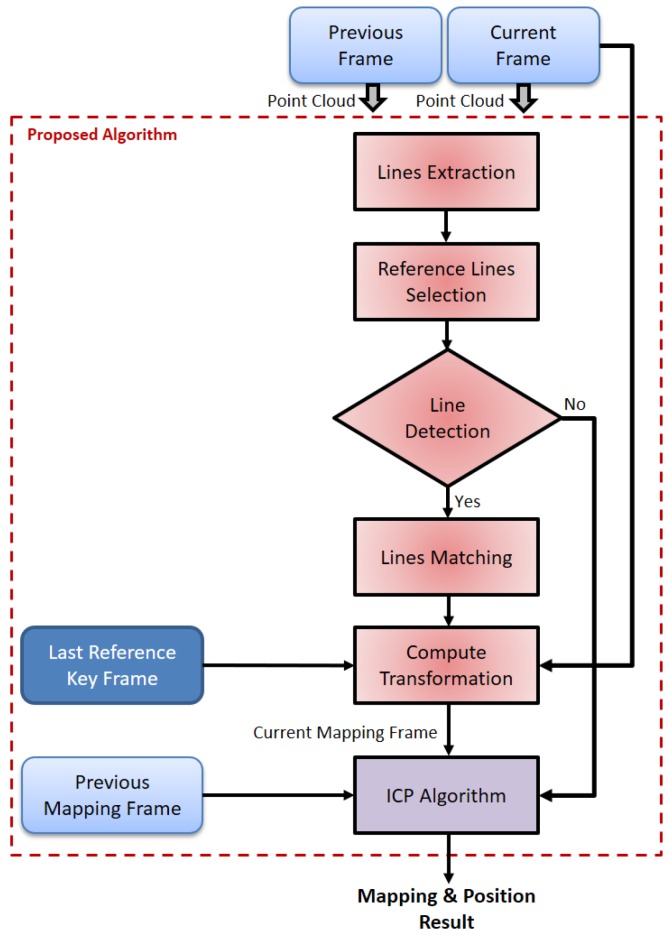
Overall structure of the proposed algorithm.

Figure 3 shows the overall structure of the proposed algorithm. The lines are extracted from every two successive scan frames. The availability of selecting two non-parallel lines as reference lines is checked. If the reference lines are detected, the lines matching process will be implemented to determine the matched reference lines in the current scan frame. Thereafter, the transformation parameters are computed with respect to the last reference key frame. After transformation to the mapping frame, the ICP algorithm is utilized with the previous mapping scan frame. Finally, the current mapping and position are computed. On the other hand, if the reference lines are not detected; the ICP algorithm is executed alone as a lines outage period.

## 3. Methodology

### 3.1. Line Extraction

**Figure 4:**
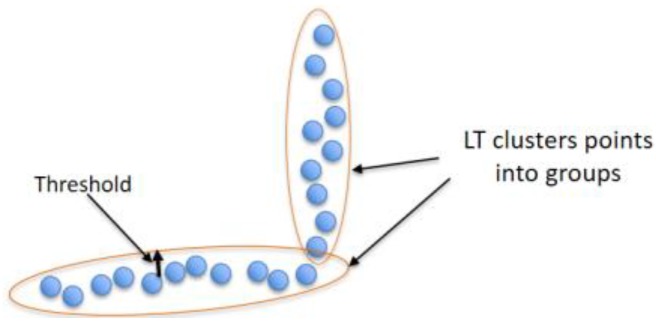
The role of line tracking (LT) algorithm.

The line-tracking (LT) algorithm [24] is used for clustering the point cloud, provided by the laser scan rangefinder, into groups per threshold $\left(T_{max}\right)$, as shown in Figure 4. This algorithm is characterized by low time complexity, an important factor in real-time systems.

The threshold $\left(T_{max}\right)$ is chosen depending on the sensor’s precision of the laser scanner range finder and the characteristics of the environment such as bricks, tiling, and painted wall. Since all the experiments are performed using a low-cost laser scanner range finder, its specifications are described in Section 4. Thus, sensor calibration process is accomplished to estimate the standard deviation (σ) of the sensor in different detection range. Table 1 lists different standard deviations of the used laser scanner range finder pursuant to the detection range. As a result of many experiments and for more confidence, it is preferable to use (2σ) in the determination of the threshold $\left(T_{max}\right)$, and adding the environment effect.

**Figure 5:**
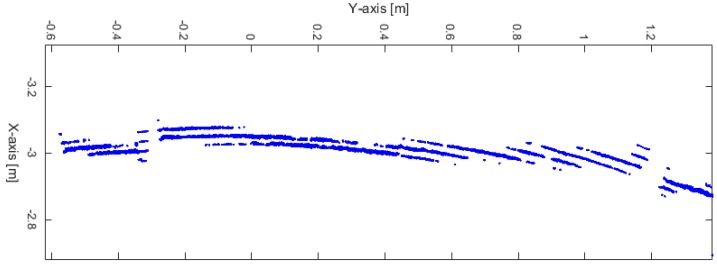
Detection behavior of the laser scanner range finder.

Figure 5 shows the detection behavior of the laser scanner range finder in a static mode for the same wall, of about 2 m in length, and from detection distance, of about 3 m. This strange behavior is due to the distance resolution and is approximately 1% of the detection distance. Therefore, the same wall detection will be represented as successive lines with a separation distance equal to the 1% of the detection distance; 3 cm in this example.

**Figure 6:**
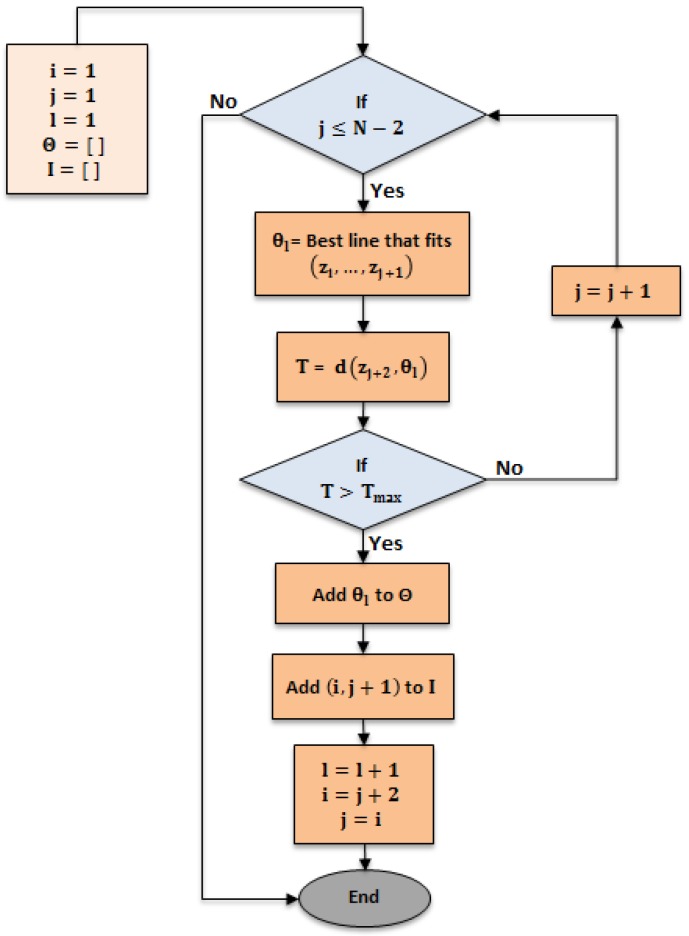
Line tracking algorithm (LT).

**Figure 7:**
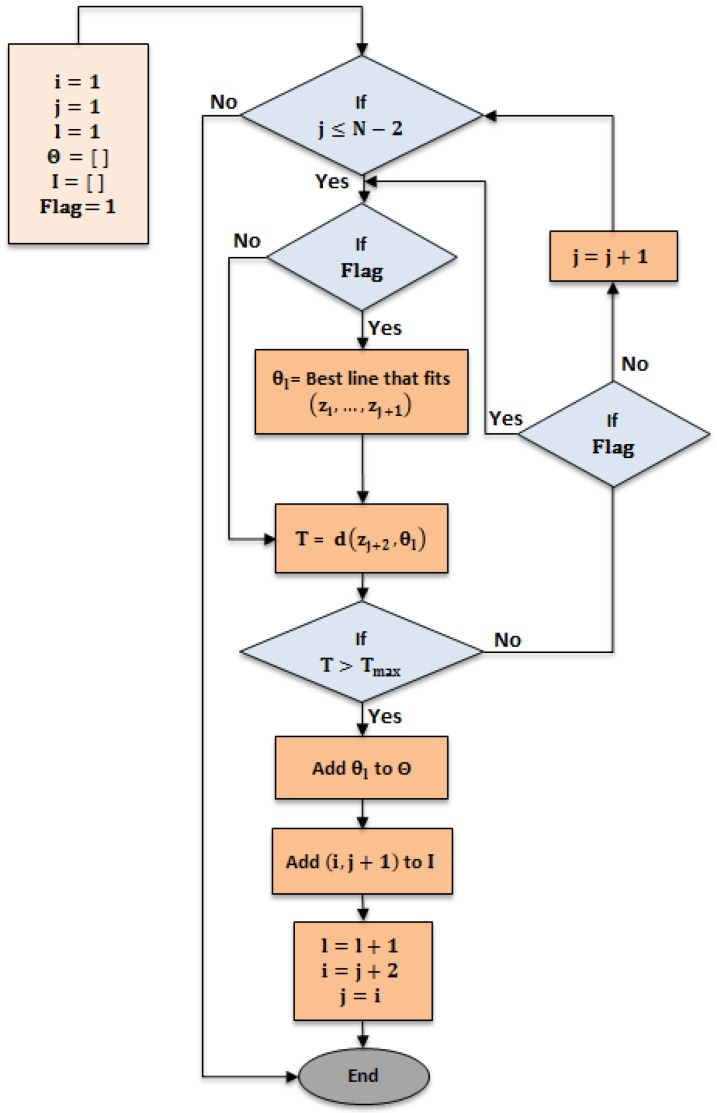
Adjusted line tracking algorithm (ALT).

Figure 6 depicts the workflow of the line tracking algorithm. The algorithm is executed in 250 ms. Figure 7 demonstrates an adjusted line-tracking algorithm for reducing time complexity down to 4 ms to be appropriate for a real-time system. The algorithm will not build a line for every new point added, unless the orthogonal distance is more than the threshold $\left(T_{max}\right)$. In this case, a new line is built from all previous points, and so the algorithm checks the threshold condition again $\left(T_{max}\right)$, and, if the condition is valid, then a new point is successively added to the previous line. If the condition is invalid, the line is terminated and a new line is started, and this is repeated until reaching the end of the dataset.

**Figure 8:**
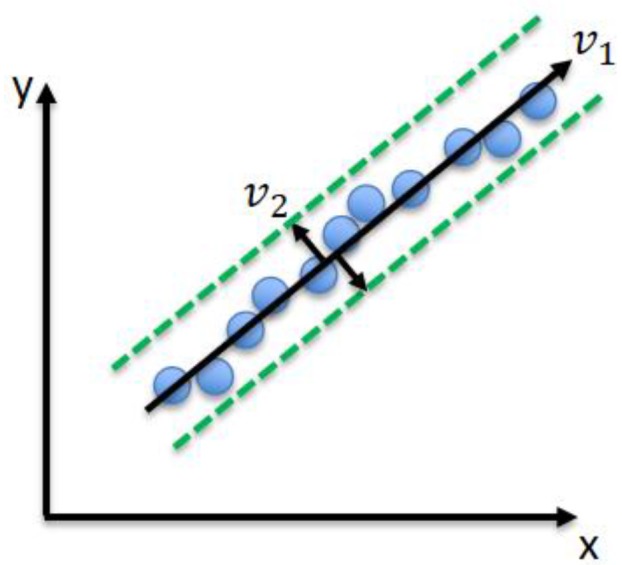
Principle component analysis (PCA).

Line fitting is implemented using principle component analysis (PCA) [25]. This statistical procedure concerns itself with the interpretation of the covariance structure of a dataset in order to identify in which principle direction the data varies. The first principle component has the largest variance, and successive principle components possess variances in descending order. From this, Eigenvalues and Eigenvectors of the covariance matrix of the data set are computed. As the data set is 2D, it has two principle components; the eigenvector $\left(v_{1}\right)$ of the biggest eigenvalue represents the principle component—the line fitting the data set, and; the second eigenvector $\left(v_{2}\right)$ represents the robustness of the line (line uncertainty), as shown in Figure 8.

**Figure 9:**
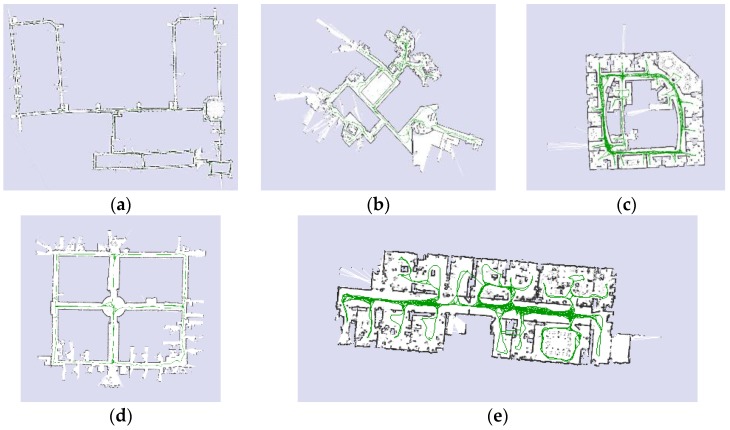
Maps for different data sets are used: (**a**) MIT Killian Court; (**b**) MIT CSAIL Building; (**c**) Intel Research Lab Seattle; (**d**) ACES Building at the University of Texas; (**e**) building 079 University of Freiburg.

The line availability in indoor environments has been evaluated using various data sets offered by different research groups. The results show that the mean number of line availability is ranging from 4.10 to 8.86 lines per scan. Figure 9 presents the tested data sets that comprise of MIT Killian Court, MIT CSAIL Building, Intel Research Lab Seattle, ACES Building at the University of Texas, and building 079 University of Freiburg, respectively [26].

**Figure 10:**
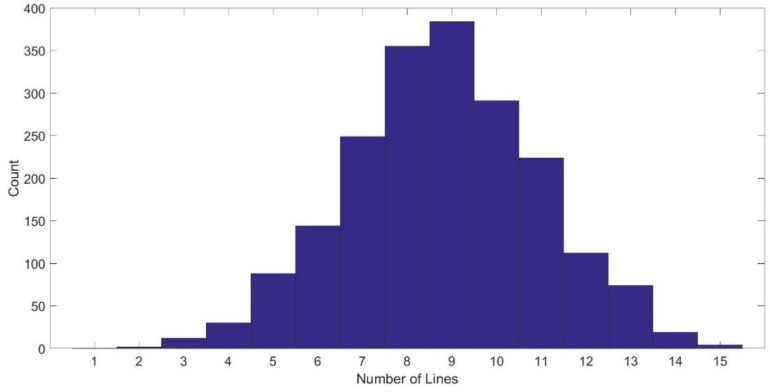
Histogram of number of lines detected in the MIT CSAIL Building dataset.

Figure 10 shows a histogram for the detected lines in the whole data set of the MIT CSAIL Building, the mean number of the detected lines is 8.8.

**Figure 11:**
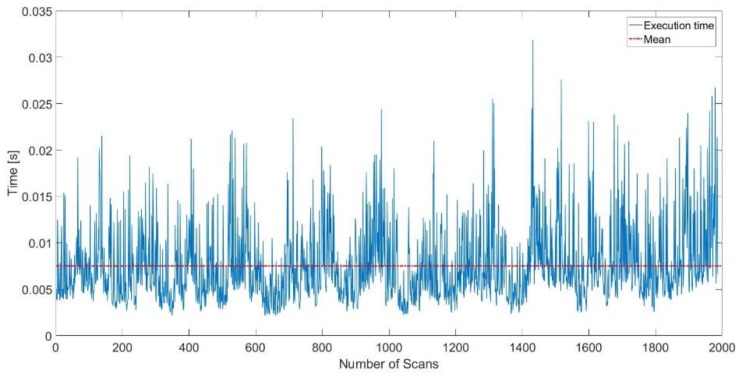
Execution time of the lines extraction in the MIT CSAIL Building dataset.

Figure 11 demonstrates the execution time of the lines extraction per scan in the MIT CSAIL Building data set, the mean execution time is 7.5 ms.

Table 2 lists the availability of lines and extraction time for different data sets. All the displayed results are for extracted lines that are composed of more than seven points.

### 3.2. Reference Key Frame

Initially, two reference lines are chosen from the extracted lines of the first point cloud, which represent the first key frame, in consonance with the following criteria: (a) the longest lines; (b) number of the points building the line is not less than the threshold $\left(min_{Pcount}\right)$; (c) the relative robustness of the lines, and; (d) the inscribed angle between the two reference lines is not less than the threshold $\left(min_{angle}\right)$.

The threshold $\left(min_{Pcount}\right)$ is selected depending on the value of the adjusted coefficient of determination $\left({\bar{R}}^{2}\right)$. Since the explanatory variables, (x, y), are fixed because the linear model is dominated for the 2D environments and although the coefficient of determination $\left(R^{2}\right)$; which is equal to the square of the correlation coefficient (r), provides an explained impression of the total variation in (y) by the linear relationship between (x) and (y). However, the coefficient of determination does not include the impact of the number of the data points that builds the line. Therefore, the adjusted coefficient of determination interprets the measure of fit considering the number of data points. The accepted percentage of the adjusted coefficient of determination is 80%:

(1) $$r=\frac{\sigma_{xy}}{\sigma_{x}\sigma_{y}}$$

(2) $${\bar{R}}^{2}=1-\left(1-R^{2}\right)\frac{n-1}{n-\left(k+1\right)}$$

where $\left(\sigma_{xy}\right)$ is the covariance between (x) and (y), $\left(\sigma_{x}\right)$ and $\left(\sigma_{y}\right)$ are the standard deviations of (x) and (y) respectively, (n) is the number of the data points, and (k) is the number of explanatory variables.

The inscribed angle $\left(min_{angle}\right)$ is chosen depending on the angle that minimize the distance of the center uncertainty $\left(d_{CenUncertainty}\right)$ of the intersection between the two reference lines. $v_{2}$ is the second eigenvector that represents the line uncertainty:

(3) $$d_{CenUncertainty}=\left|\frac{v_{2}}{\sin(min_{angle})}\right|$$

**Figure 12:**
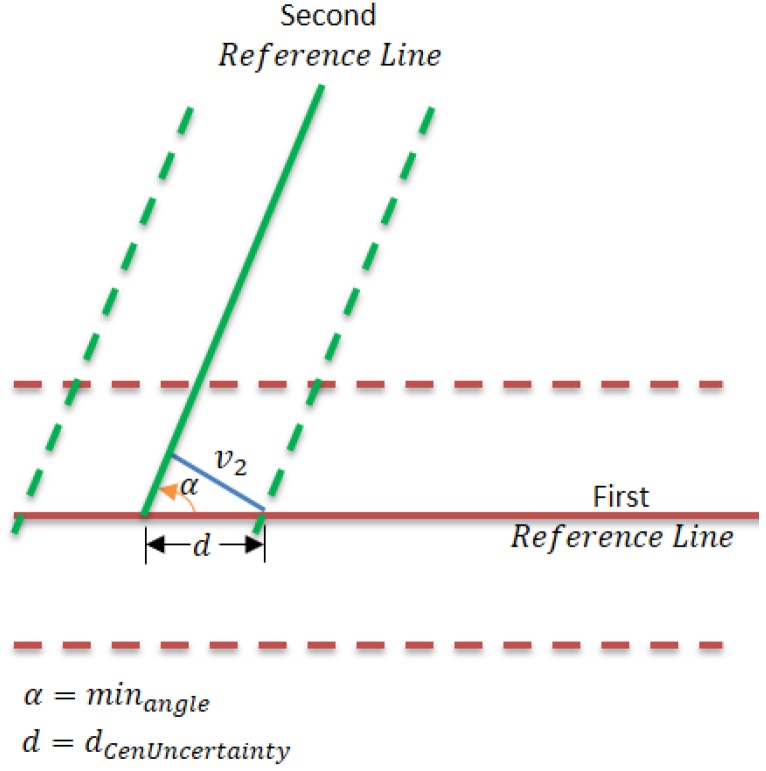
Setting min angle between two reference lines.

Figure 12 shows the inscribed angle between the two reference lines and its impact on the distance of the center uncertainty. The red solid line represents the first reference line while the red dashed lines present the line uncertainty. On the other hand, the green solid line represents the second reference line and the green dashed lines represent the line uncertainty. The blue line represents the uncertainty distance (second eigenvector) of the second reference line. The distance of the center uncertainty will positively affect the selection of the two reference lines. Since the algorithm depends on two non-parallel reference lines, assigning the min accepted inscribed angle depends on the environment structure and the precision of the range finder sensor.

**Figure 13:**
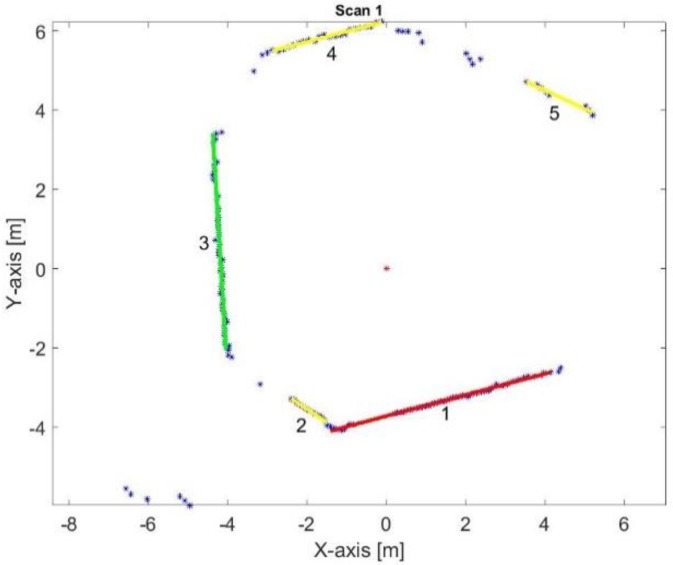
Two reference lines of the first key frame.

Figure 13 illustrates the two reference lines of the first key frame. The main reference line is represented by the red color and the green line is the second reference line, while the yellow color presents the rest lines. The position of the laser range finder is represented by the red asterisk.

Table 3 lists the computed coefficient of determination, adjusted coefficient of determination, number of data points that build each extracted line in the scan, and length of the extracted lines in the first key frame as shown in Figure 13. Line number (1) is chosen to be the first reference line because it is the longest line, most robust line, and is built from the max points group. Although line number (3) is selected to be the second reference line and this line is not the second robust line, but it is the second longest line and is built from the second max points group, and further, it exceeds the min accepted percentage of the adjusted coefficient of determination which is 80%.

**Figure 14:**
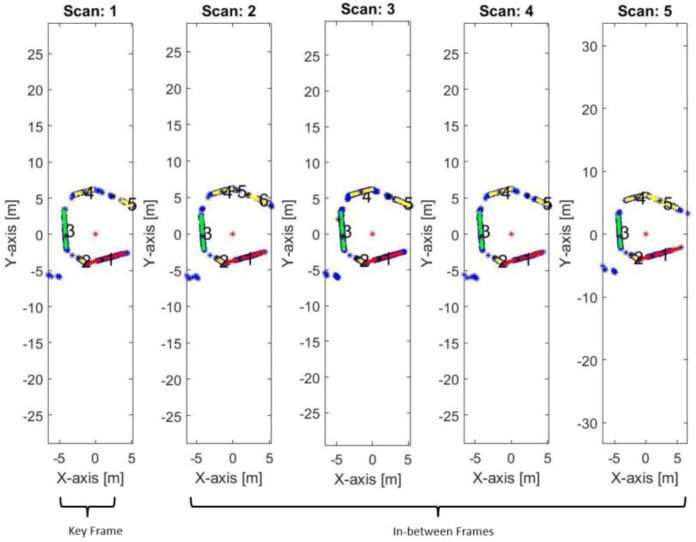
The first key frame and the successive in-between frames.

The first two reference lines are the kernel for the mapping frame. Afterward, all the in-between frames until receiving another key frame are sharing in constructing a coherent map using their directly relation with the reference key frame, as shown in Figure 14.

The vehicle’s absolute position with respect to the reference lines in the mapping frame is determined by calculating the orthogonal distances from it to the reference lines at every period. This helps compensate for any potential accumulated errors, especially given that other techniques for determining relative position [27] have difficulties with error accumulation over time. Orthogonal distances from the vehicle position to all the lines are calculated as well, and additionally, the intersection points between the orthogonal lines and all the lines are computed.

### 3.3. Scan Matching

Representing each scan using a group of lines is more reliable than using a group of points because correct data association for each point between two successive scans is quite a difficult process. In contrast, matching lines is both reliable and robust. A line is considered to be matched after accepting mutual compatibility with the previous scan line. Matching criteria, described hereafter, are performed according to several computations, thereby ensuring a correct match. Thence, angles between the previous scan lines and all current lines are computed using a vector dot product. Candidate lines whose angle is less than a certain threshold $\left(max_{ScanRotationAngle}\right)$ are selected. The threshold $\left(max_{ScanRotationAngle}\right)\;$is opted according to the max rotation angle of the vehicle based on its dynamic. Subsequently, the candidate line achieving the following two conditions is selected: (a) it possesses the smallest orthogonal distance compared with the previous one, and; (b) it possesses the smallest Euclidean distance of the current intersection points with the previous one. Finally, if the smallest orthogonal distance is bigger than the threshold $\left(max_{OrthogonalShift}\right)$, there is no matching, otherwise, matching does occur. The $\left(max_{OrthogonalShift}\right)$ is selected depending on the max speed of the vehicle based on its dynamic as well.

**Figure 15:**
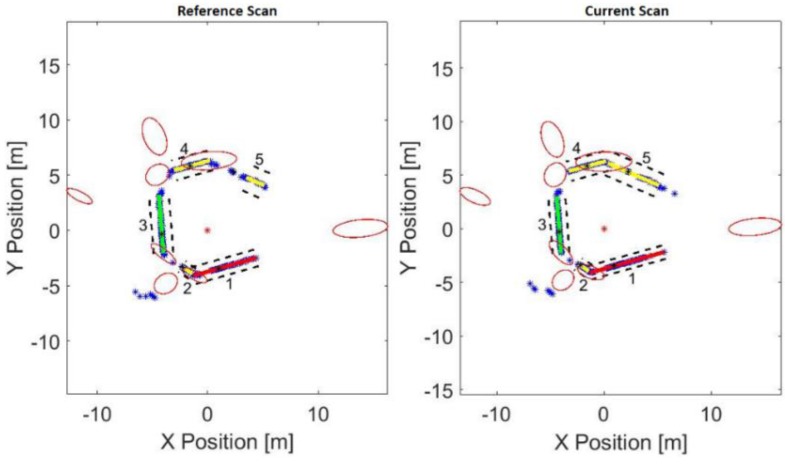
Corners detection and its confidence ellipsoid region.

After the lines are matched, the new matched reference lines become the new reference lines and a new position for the vehicle is determined. Thereafter, the angles between all matched lines in each scan frame are computed. Using a specified threshold $\left(min_{angle}\right)$ angle, lines accepting this threshold are selected, and intersection points for the selected lines are then calculated regardless of the physical intersection of these line segments in the scan. These, then, become the corners for both previous and current environments. To account for inherent uncertainties within detected corners, the covariance of the corners is estimated using extracted line variances, as illustrated in Figure 15. The red ellipses present the confidence ellipse regions for each detected corner, while the black dashed lines present the precision of the lines that built around the matched lines using the second Eigenvalue. The intersection points, between the position of the laser range finder and each detected line, are represented by the black asterisks.

**Figure 16:**
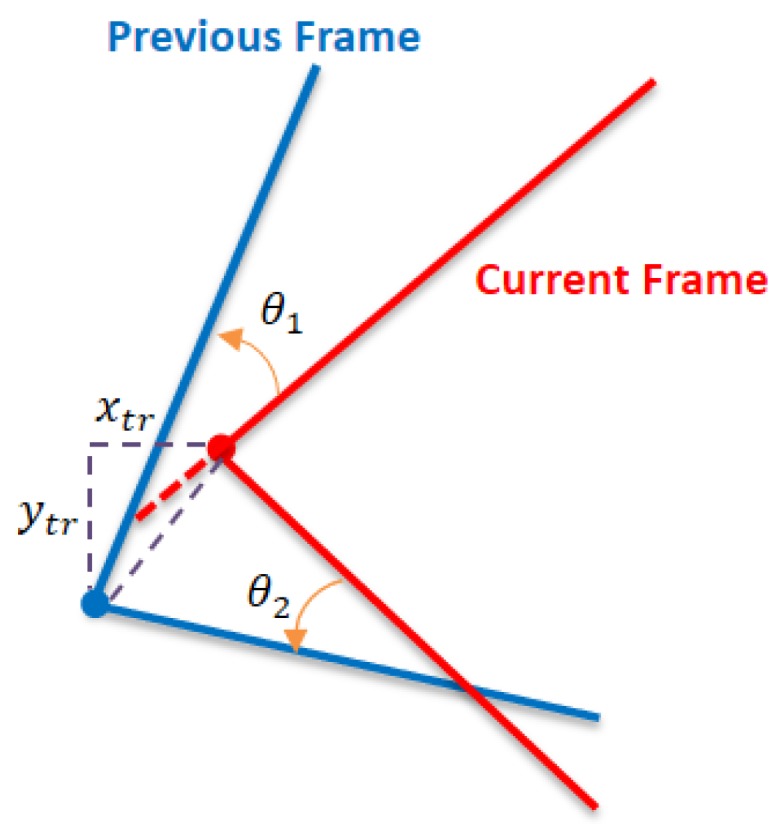
Line registration algorithm.

Using least squares, the detected corners are used for estimating transformation parameters $\left(\theta ,\;x_{tr},\;y_{tr}\right)\;$between successive scans. These parameters are used to calculate an adjusted initialization for the scan matching process. This method can be employed to match successive scans, but it can also be used to support other iterative methods for achieve a more effective and faster convergence. However, the detected corners sometimes decreased to be only one corner. Therefore, the corners registration fails to compute the transformation parameters. Figure 16 demonstrates the line registration algorithm using two non-parallel lines with one corner. The required rotation (θ) is the angle that minimizes:

(4) $$E\left(\theta \right)=argmin\;\overset{2}{\underset{i=1}{\sum }}\left|S_{pi}-S_{ci}\right|$$

where $\left(S_{p}\right)\;$and $\left(S_{c}\right)$ are the slope of the previous and current lines respectively. While the translation $\left(x_{tr},\;y_{tr}\right)$ is computed from distance that minimizes the range between the detected corners in the successive scans. Consequently, an adjusted corners registration is proposed to estimate the transformation parameters using corners registration and further line algorithm for one corner condition.

The ICP is an algorithm employed for finding the transformation between two sets of point clouds by minimizing the difference between them. In case of a 2D data set, the transformation possesses three degrees of freedom (i.e., a combination of translation and rotation). The primary challenge for the ICP algorithm is determining the correct data association between the two point clouds. While the ICP algorithm encounters problems in data association during sharp rotation and/or fast movement, the corners registration method helps the ICP improve data association even in harsh situations [28]. Occasionally there are no new references matched with previous ones, and in this case, swapping to new reference lines occurs, creating new reference key frame.

### 3.4. Successive Key Frame

After a while the reference lines lessen due to the movement of the vehicle. In order to preserve continuity of the reference lines occurrence, the successive key frames are created when the length of the reference lines reaches a threshold. The goal here is to locate two new reference lines in the previous scan frame, and matched lines in the current scan frame, while preserving the chosen line criteria using the algorithm as outlined in Section 3.2. The transformation matrix between the old reference lines and the new reference lines in the previous scan frame is then computed, and from this, the transformation matrix from the new matched reference lines in the current scan frame and the new reference lines in the previous scan frame, can be determined. Additionally, the vehicle’s relative current position to the new matched reference lines in the current scan frame is computed.

**Figure 17:**
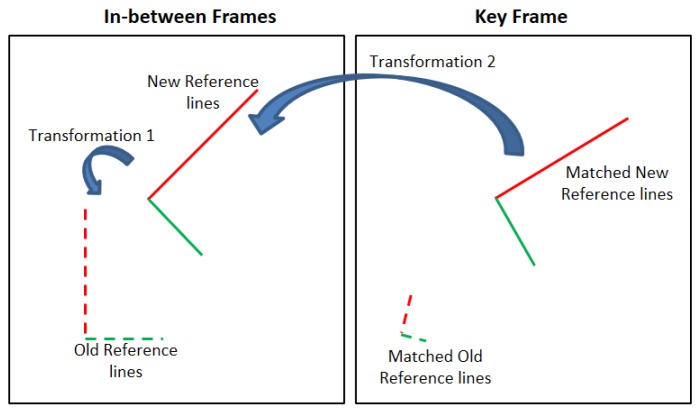
Swapping process between the old and new reference lines.

Figure 17 illustrates the swapping process between the old reference lines of the first key frame and the new reference key frame, this process is formalized in Algorithms 1 and 2 as well. The old reference lines of the first key frame are represented by the dotted lines either in the in-between frames and the key frame, while the solid lines present the two new reference lines. Transformation 1 is the relation between the old reference lines of the first key frame and the two new reference lines in the in-between frames. Transformation 2 is the transition between the two matched reference lines in the in-between frames and the key frame.

**Algorithm 1:** Pseudo-code of the swapping process between the old and new reference lines.1:**Setting:** sort the previous lines in descending order according to their length2:**For** each previous line from long to short3:   **If** this line has matching line in the current scan and composes from number of points more than threshold4:      calculate the unit vector of this line (1st reference)5:      for each previous rest line from long to short6:       **If** this line has matching line in the current scan and composes from number of points more than threshold7:          calculate the unit vector of this line (2nd reference)8:          calculate the intersected angle between the two vectors9:          **If** angle > 9010:            angle = 180−angle11:          **End if**
12:          **If** absolute angle > threshold13:            swap the two old reference lines with the new ones14:            raise flag15:          **End if**
16:        **End if**
17:      **End for**
18:  **End if**
19:**End for**

**Algorithm 2:** Pseudo-code of computing the relation between the old and new reference lines.1:**If** flag is high2:  calculate the unit vector of the old 1st reference line3:  calculate the unit vector of the new 1st reference line4:  compute the intersected angle between the two vectors5:  compute the cross product of the two vectors6:  **If** the 3rd component of the cross-product result is less than zero7:      angle = −angle8:  **End if**
9:**End if**

**Figure 18:**
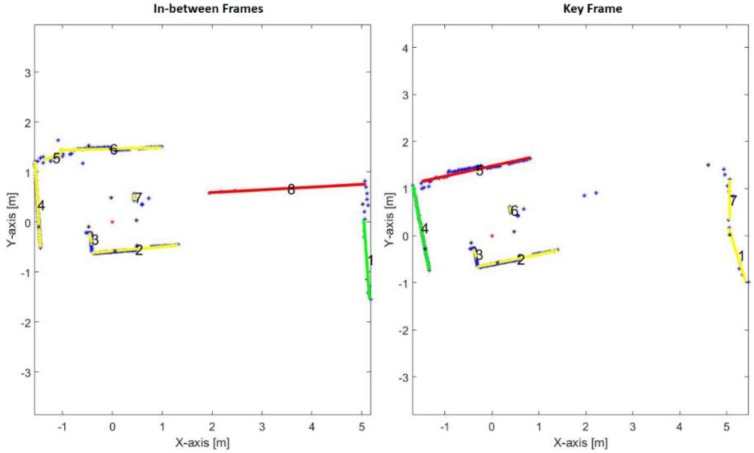
Swapping result between old and new reference lines.

Figure 18 demonstrates the swapping result between the old reference lines of the first key frame and the new reference key frame. It is obvious that the first old reference line, line number 8 in the in-between frame, does not exist in the next frame. Therefore, a new key frame must perform in order to select new reference lines for the next frames. The new first and second reference lines are numbered by 5 and 4 respectively, in the key frame.

### 3.5. Iterative Closest Point (ICP)

Finally, the ICP algorithm is used to approximate the current adjusted scan’s point cloud to the previous one in the mapping frame in order to fine tune the mapping, and accurately determine the vehicle’s new position. Using this algorithm by itself is problematic for two reasons: (a) the vehicle can get lost in the case of rapid movement or sharp rotation, and; (b) the ICP algorithm uses an iterative technique, and so requires significant amounts of time for scan matching convergence.

**Figure 19:**
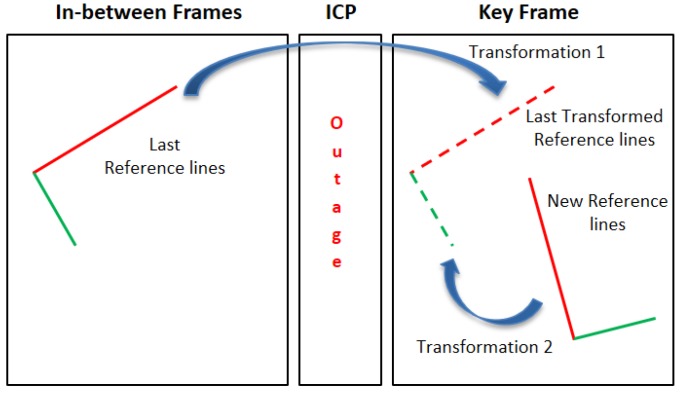
Last reference lines transformation after the lines outage period.

On the other hand, the ICP algorithm is solely performed during the lines outage period. During this period the transformation parameters, in the laser scanner coordinate frame, are cumulatively computed besides the transformation parameters of the global map. When the lines are detected over again, the last reference lines are transformed to the current scan frame using the computed laser scanner transformation. Thereafter, the relation between the new reference lines and the transformed reference lines are calculated to overcome the reference lines discontinuity during the lines outage period as shown in Figure 19.

## 4. Experimental Results

**Figure 20:**
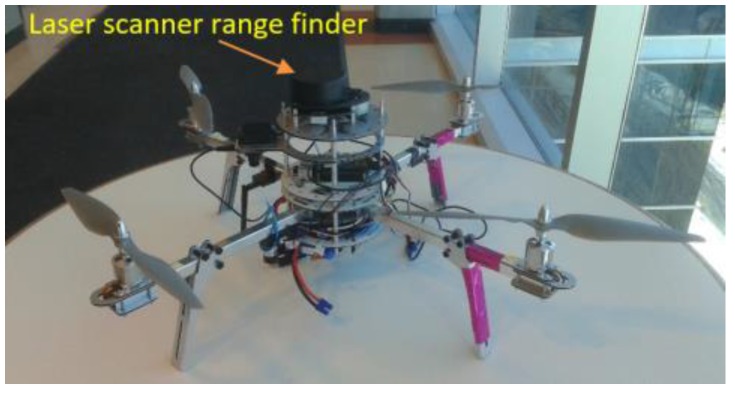
The aerial platform equipped with the laser scanner range finder.

All the experimental results are performed using a low-cost laser scanner range finder (RPLIDAR 360°, SlamTec, Zhangjiang, Shanghai, China) to reduce the cost of the system. This laser range finder is characterized by approximately short detection range, 6 m, max scan rate (rotation speed), 7 Hz, and angular resolution at the max rotation speed, 1.5°. Figure 20 presents the aerial platform equipped with the laser scanner range finder. All the datasets are collected in manual mode.

For the sake of comparison between different algorithms, all algorithms have been implemented using the same computing platform (MATLAB) on a Lenovo ThinkPad, Intel core i7-4702MQ 2.2 GHz, 4G RAM, and 64-bit operating system. In order to validate and evaluate the proposed algorithm, the mapping performance and time consumption of the proposed algorithm are compared with Hector SLAM with different grid cell dimensions [29], iterative closest point (ICP), and feature to feature registration such as corners, in static and dynamic environments. The environments include, but not limited to, glass objects, bricks, longer corridors than the max detection range of the laser scanner and aluminum curtains to create harsh scenarios as described in Table 4.

### 4.1. Static Environment

#### 4.1.1. Dataset I

**Figure 21:**
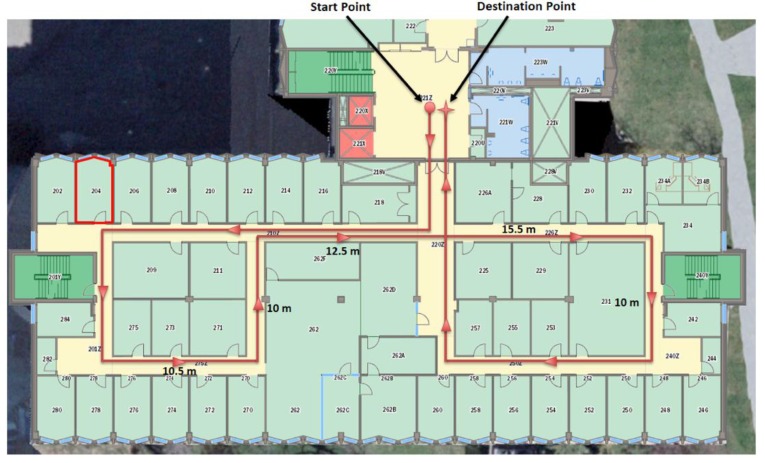
Map representation of the dataset I.

Figure 21 represents the environmental structure of the dataset I; ENF building at University of Calgary, and the performed trajectory is represented by the red line. The red circle presents the start point of the trajectory and the red star represents the final destination of the trajectory. All the corridors lengths are longer that the max detection range of the laser rangefinder.

**Figure 22:**
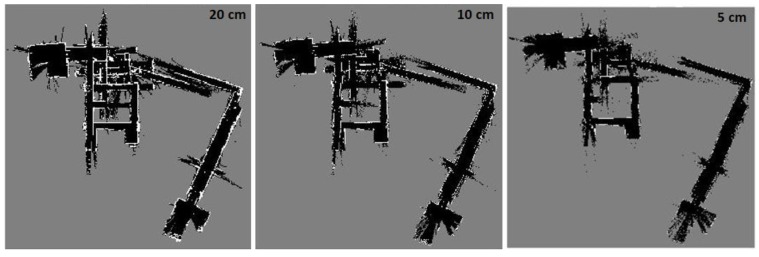
Hector SLAM three level multi-resolution map representation result using grid cell dimensions 20, 10 and 5 cm.

Figure 22 illustrates Hector SLAM three level multi-resolution map representation result using grid cell dimensions 20, 10 and 5 cm. Due to the existence of the long corridors, Hector SLAM fails to estimate the longitudinal movement. Thus, it accumulates the point cloud of the successive scans approximately at the same position. As a result, it fails to converge and build a representation for the environment.

**Figure 23:**
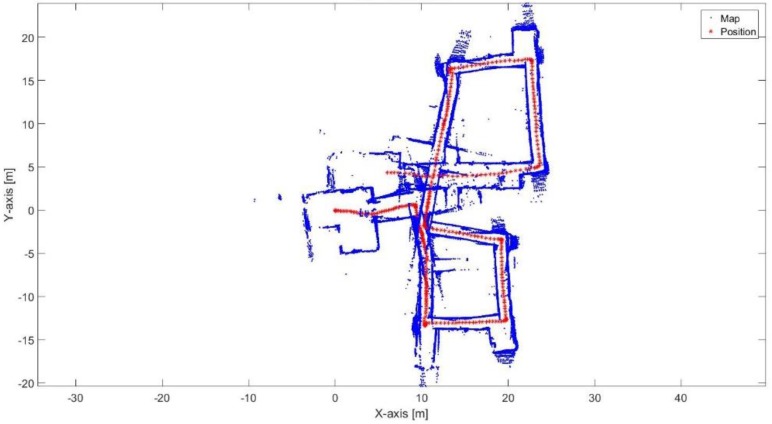
Mapping and position results for the ICP algorithm.

Figure 23 shows the mapping and position results using ICP algorithm. The red points present the implemented trajectory. The ICP algorithm fails to correctly represent the environment because of the corridors as well. Furthermore, it fails to determine the orthogonality behavior of the corners, this is due to the aggressive maneuver causes incorrect estimation of the rotation parameter.

**Figure 24:**
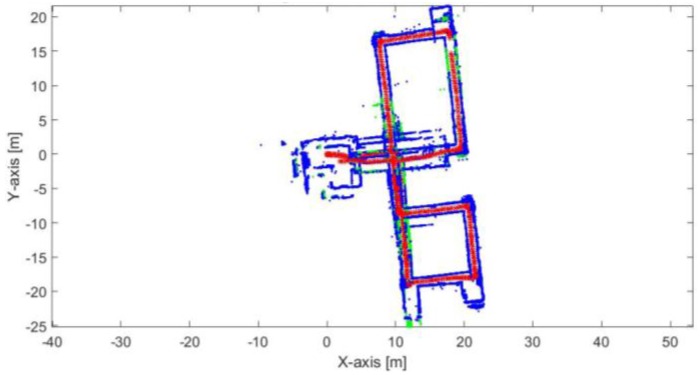
Mapping and position results for the proposed algorithm.

Figure 24 demonstrates the mapping and position results using the proposed algorithm alone without using loop closure and/or external sensors. The blue points represent the mapping result during the RKF method while the green points represent the mapping result using the ICP algorithm during the lines outage period. The red points present the implemented trajectory. Although the existence of the corridors and aggressive maneuvers, it is obvious that the proposed algorithm succeeds to converge and build a map of the environment. It also achieves to represent the orthogonality behavior of the corners. The shrink in some corridors is due to the dependence of the ICP algorithm alone during the lines outage period. The mean execution time is 7 ms.

#### 4.1.2. Dataset II

**Figure 25:**
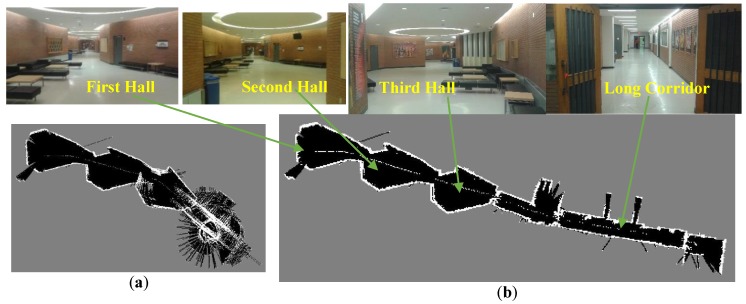
Single level hector SLAM mapping results using different grid cell dimensions: (**a**) 10 cm; (**b**) 20 cm.

Dataset II is collected at ENE building University of Calgary. Hector SLAM completely fails to converge and build a map when using single level with grid cell dimension equal to 5 and 10 cm because the algorithm is stuck in local minima as it is based on gradient ascent. However, it succeeds with higher cell dimension such as 20 cm and more, as shown in Figure 25.

**Figure 26:**
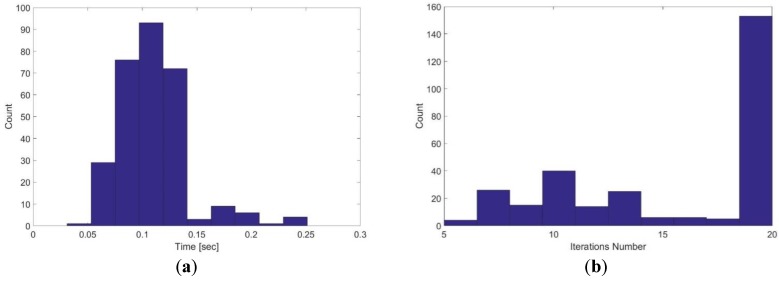
Single level hector SLAM using 20 cm grid cell dimension: (**a**) Execution time; (**b**) iterations number.

Figure 26 shows the execution time and the iterations number for single level hector SLAM using 20 cm grid cell dimension. The mean execution time is 0.11 s while the mean iterations number is 15.7.

**Figure 27:**
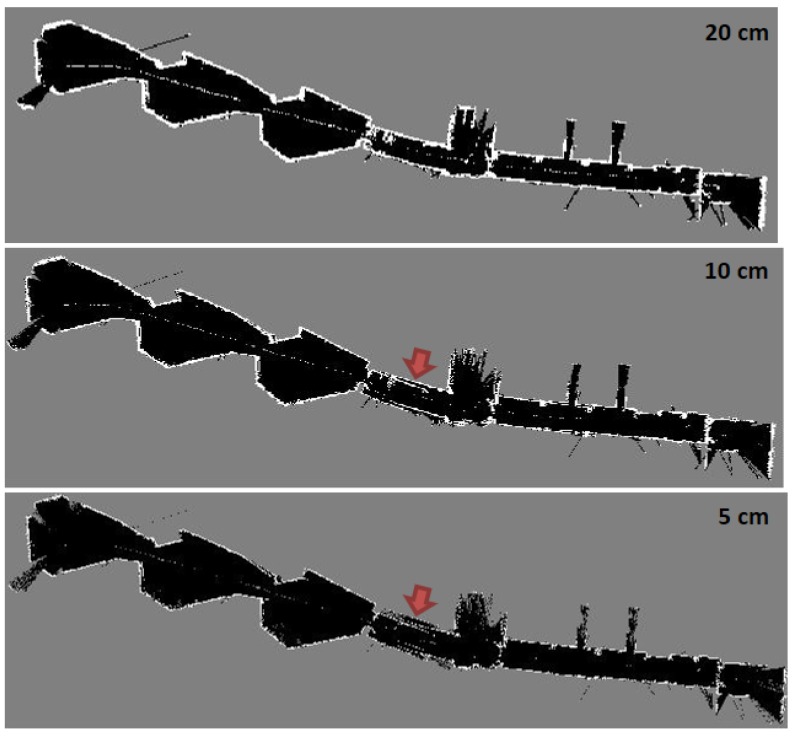
Three level multi-resolution map representation result using grid cell dimensions 20, 10 and 5 cm.

Using Hector SLAM with single level grid cell dimension is potentially apt to get stuck in local minima. Therefore, multi-resolution map representation is used to mitigate this problem [28]. However, these multiple map levels are memory and time consuming because they are keeping different map levels in memory and simultaneously updating them, furthermore, each level takes many iterations in order to converge. Figure 27 shows the mapping result of three level multi-resolution map representation using grid cell dimensions 20, 10 and 5 cm. Although the high grid level aids the low grid level to converge and build the entire map compared with Figure 25a but it also fails to converge in different parts as presented by the red arrows in Figure 27.

**Figure 28:**
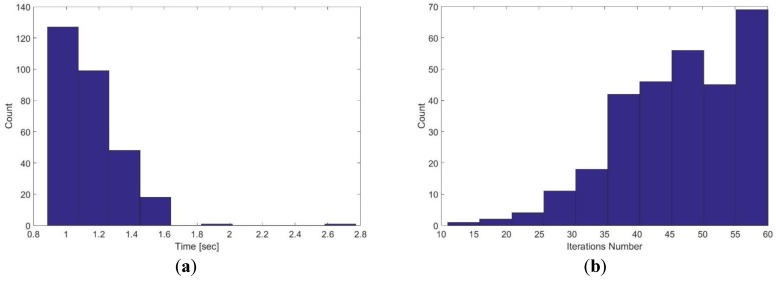
Execution time and iterations number for three level multi-resolution map representation with 20, 10 and 5 cm grid cell dimensions. (a) Execution time; (b) iterations number.

Figure 28 demonstrates the execution time and iterations number for three level multi-resolution map representation with 20, 10 and 5 cm grid cell dimensions. It is obvious that the time consumption and number of iterations are high compared with the single level grid cell dimension (1.15 s and 47 respectively). Moreover, the processing time for the multi-resolution map representation is not adequate for real-time systems.

Table 5 shows the mean execution time and number of iterations for multi-resolution map representation with different levels and grid cell dimensions.

**Figure 29:**
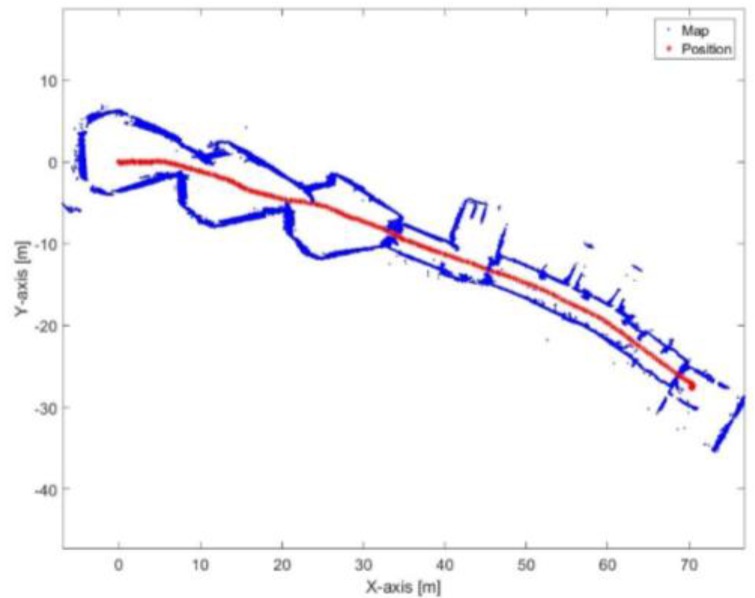
Mapping and position results for the ICP algorithm.

**Figure 30:**
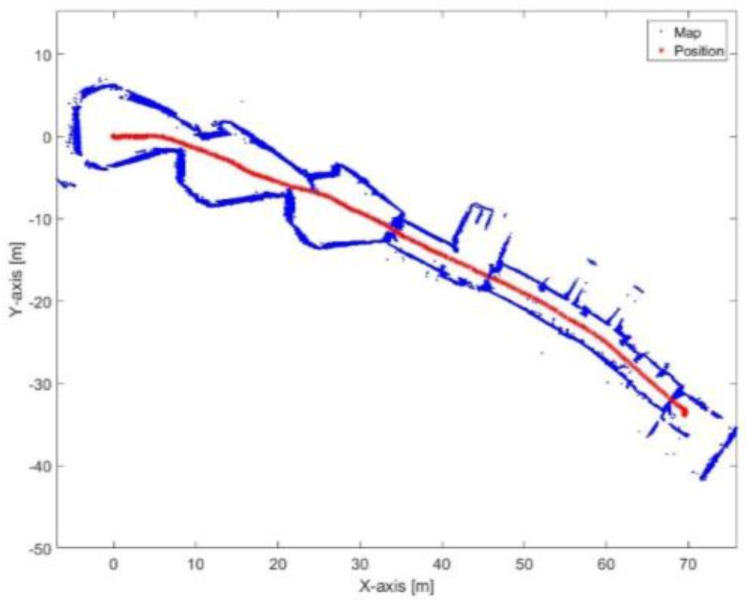
Mapping and position results using adjusted corners registration.

Figure 29 and Figure 30 show the mapping and position results using ICP algorithm and adjusted corners registration respectively. The red asterisk represents the trajectory of the vehicle. The ICP algorithm succeeds to assign good correspondences because there are no aggressive manoeuvers. The corners registration fails because sometimes the algorithm does not find two corners while the adjusted corners registration succeeds. The mean execution time and number of iterations for the ICP algorithm are 13 ms and 20, respectively, while the corners registration results for the mean execution time and number of iterations are 9 ms and 17, respectively. The performance of the generated map is sensitive to the maximum range detection of the sensor [30]. It is clear that both algorithms were prone to bending in the corridor. However, they are still capable for vehicle’s navigation because the generated map maintains a correct topology of the environment by reflecting the spatial structure of the corridor.

**Figure 31:**
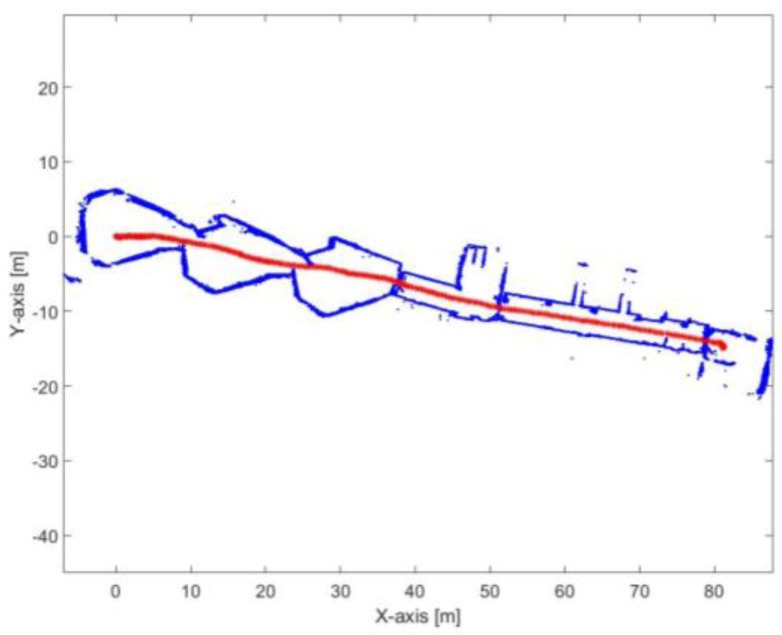
Mapping and position results for the proposed algorithm.

Figure 31 presents the mapping and position results using the proposed algorithm. The mean execution time and number of iterations for the proposed algorithm are 9 ms and 13.46, respectively. It is obvious that the bending in the corridor is almost vanished because the proposed algorithm depends on the transformation parameters between the reference lines of the current scan frame with respect to the first reference key frame each time. Thus, if the transformation was incorrectly estimated in t epoch, this would not affect the estimated transformation of t+1 epoch. Furthermore, the processing time of the proposed algorithm is proper for real-time systems.

#### 4.1.3. Dataset III

**Figure 32:**
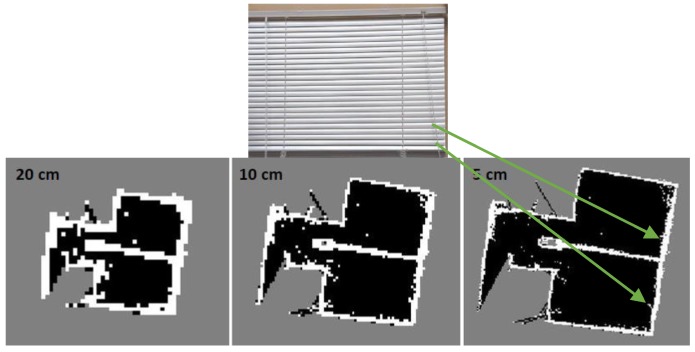
Three level multi-resolution map representation result using grid cell dimensions 20, 10 and 5 cm.

Figure 32 illustrates three level multi-resolution map representation result using grid cell dimensions 20, 10 and 5 cm. The mean execution time and number of iterations are 0.9 s and 38.35, respectively.

**Figure 33:**
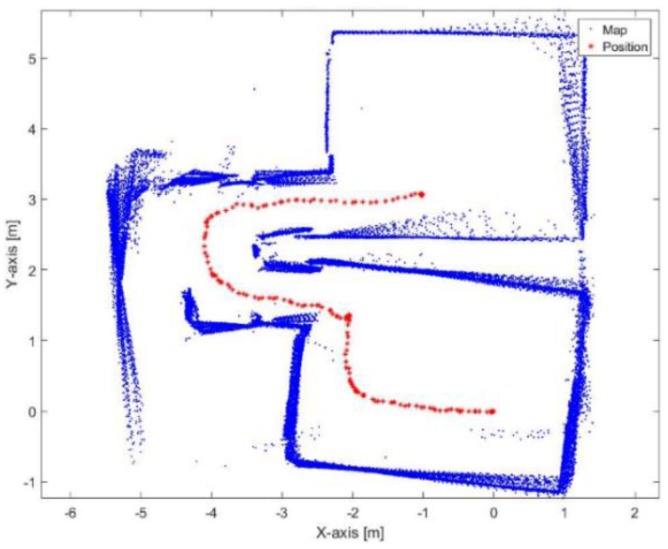
Mapping and position results for the ICP algorithm.

**Figure 34:**
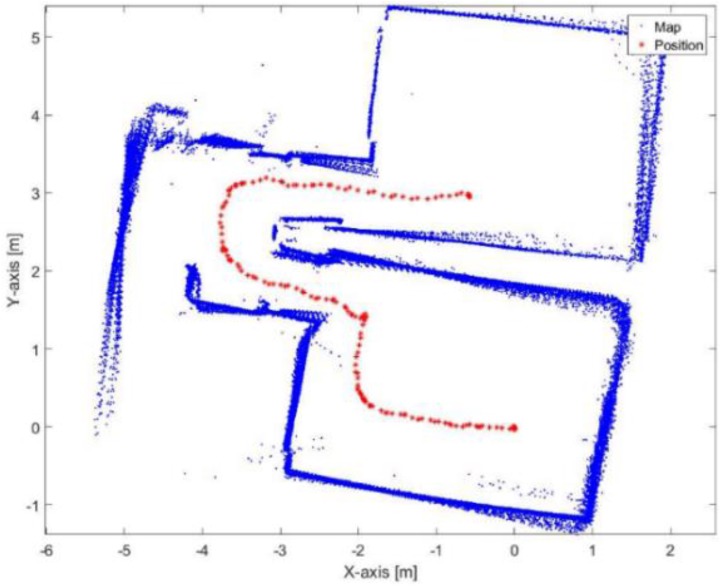
Mapping and position results for the adjusted corners registration.

Figure 33 and Figure 34 show the mapping and position results using ICP algorithm and adjusted corners registration respectively. The red asterisk represents the trajectory of the vehicle. Both algorithms fail to assign good correspondences during the sharp rotation period. The mean execution time and number of iterations for the ICP algorithm are 14 ms and 20, respectively, while the corners registration results for the mean execution time and number of iterations are 10 ms and 17, respectively.

**Figure 35:**
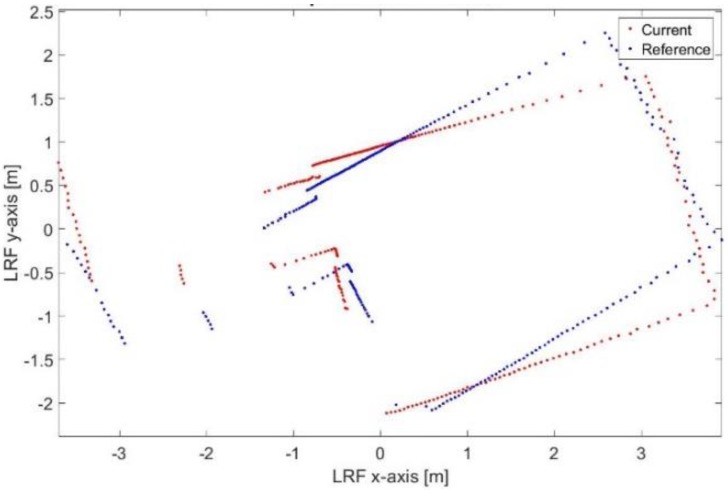
Failure of the proposed scan matching algorithm in one of the in-between frames w.r.t the successive frame.

**Figure 36:**
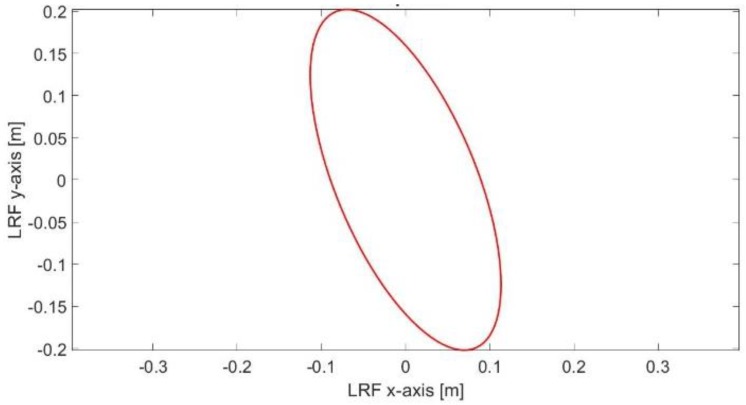
One sigma ellipse of the scan matching error.

**Figure 37:**
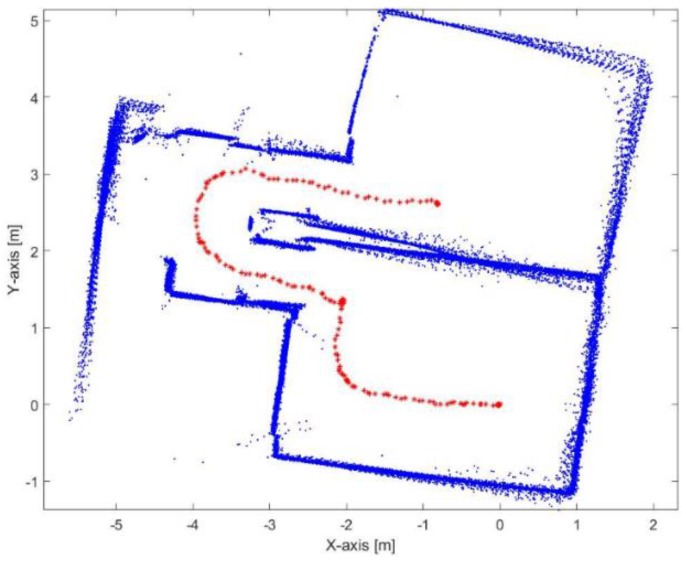
Mapping and position results for the proposed algorithm.

Figure 35 shows the scan matching result, between two successive scan frames, during aggressive maneuver where the ICP algorithm has been trapped in local minima. The matching RMSE between the matched points is 23.33 cm. Figure 36 shows the scan matching one (σ) error ellipse. However, such misalignment has been corrected in the final solution using the proposed RKF approach as shown in Figure 37.

Figure 37 presents the mapping and position results using the proposed algorithm. The mean execution time and number of iterations for 9.3 ms and 13.5, respectively.

#### 4.1.4. Dataset IV

**Figure 38:**
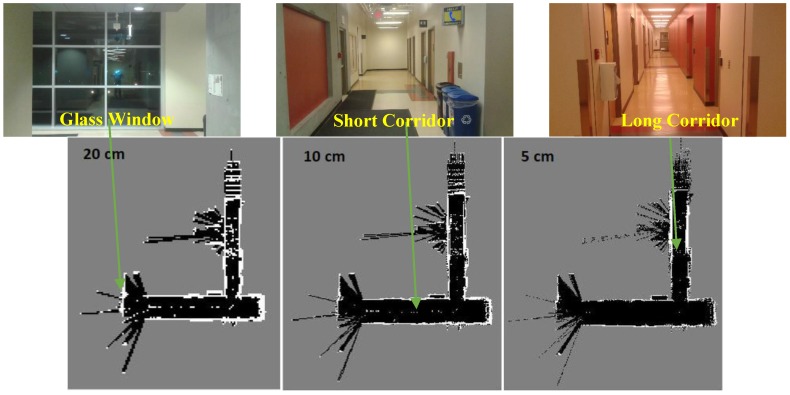
Three level multi-resolution map representation result using grid cell dimensions 20, 10 and 5 cm.

Figure 38 illustrates three level multi-resolution map representation result using grid cell dimensions 20, 10 and 5 cm, the dataset is collected at the University of Calgary CCIT building. The mean execution time and number of iterations for 0.94 s and 48.4, respectively.

**Figure 39:**
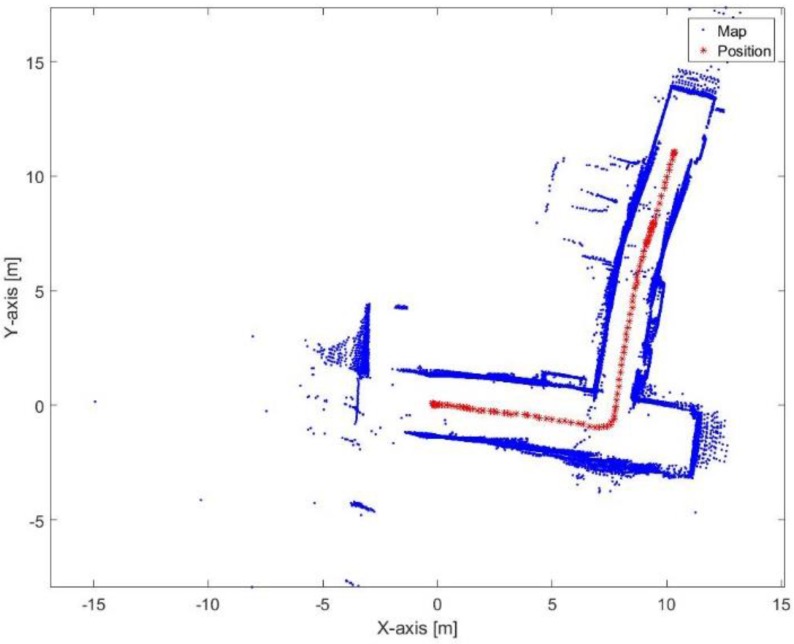
Mapping and position results for the ICP algorithm.

**Figure 40:**
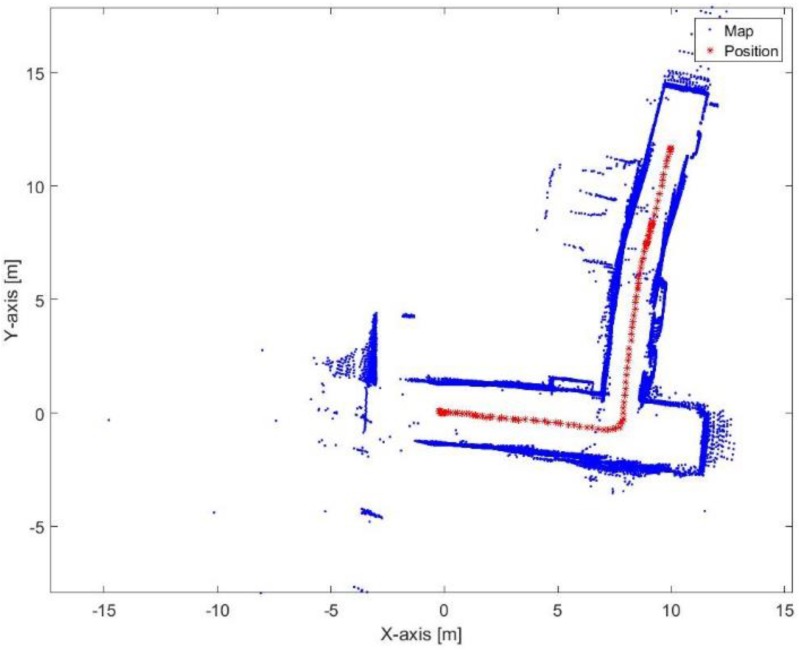
Mapping and position results for the adjusted corners registration.

Figure 39 and Figure 40 show the mapping and position results using ICP algorithm and adjusted corners registration respectively. The red asterisk represents the trajectory of the vehicle. Both algorithms fail to assign good correspondences during the sharp rotation period. The mean execution time and number of iterations for the ICP algorithm are 9 ms and 20, respectively, while the corners registration results for the mean execution time and number of iterations are 9 ms and 17, respectively.

**Figure 41:**
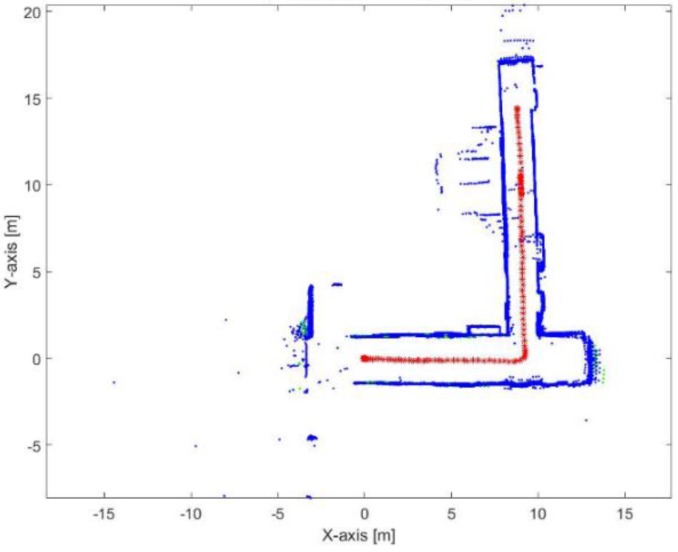
Mapping and position results for the proposed algorithm.

Figure 41 presents the mapping and position results using the proposed algorithm. The mean execution time and number of iterations for 8.9 ms and 13.5, respectively.

### 4.2. Dynamic Environment

#### Dataset V

**Figure 42:**
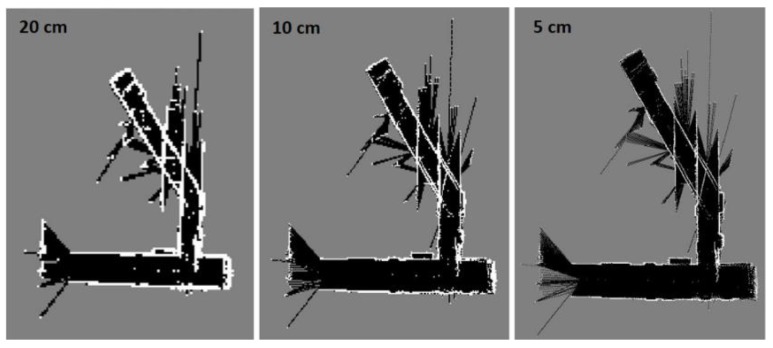
Three level multi-resolution map representation result using grid cell dimensions 20, 10 and 5 cm.

Figure 42 depicts three level multi-resolution map representation result in dynamic environment using grid cell dimensions 20, 10 and 5 cm. The Hector SLAM does not afford the noise cells that arise from the moving objects. Thence, the Hector SLAM fails to converge and construct the map. The mean execution time and number of iterations for 1.2 s and 48.3, respectively.

**Figure 43:**
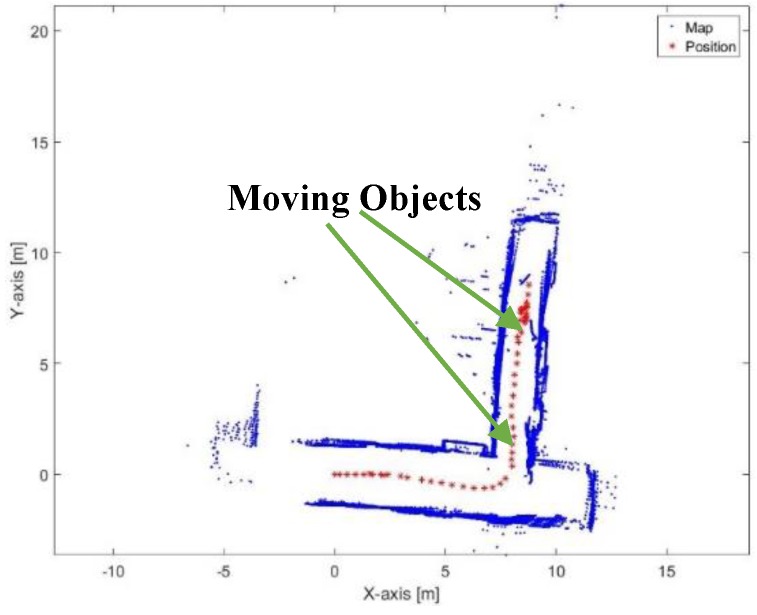
Mapping and position results for the ICP algorithm.

**Figure 44:**
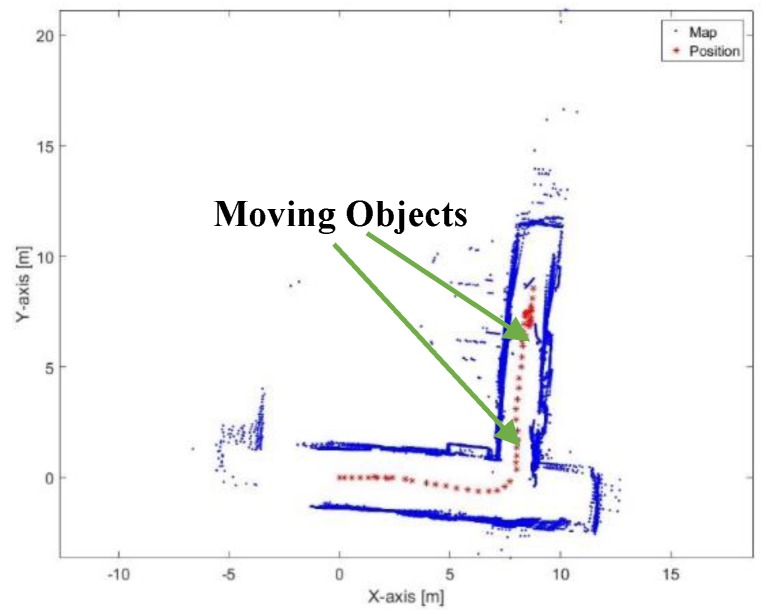
Mapping and position results for the adjusted corners registration.

Figure 43 and Figure 44 show the mapping and position results using ICP algorithm and adjusted corners registration respectively. Although, both algorithms succeed to converge, that they have a scale problem, which is clear in the second corridor. The bad data association due to the moving objects affects the solution in both algorithms.

**Figure 45:**
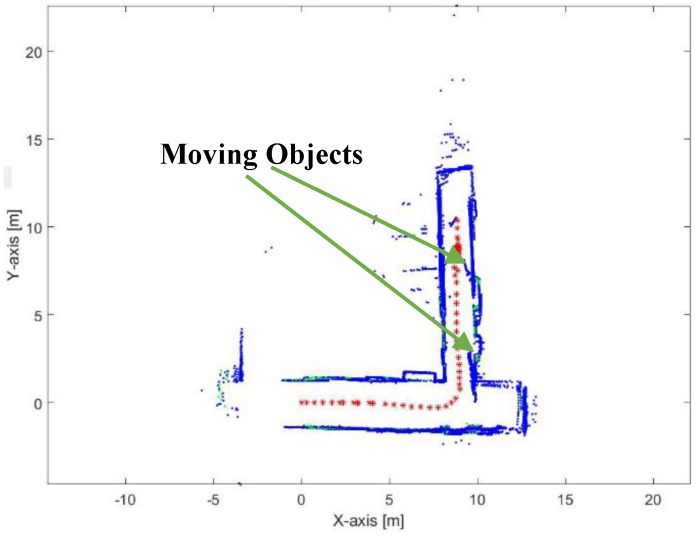
Mapping and position results for the proposed algorithm.

Figure 45 presents the mapping and position results using the proposed algorithm. The proposed algorithm does not suffer from the moving objects because the extracted lines from the moving objects does not accept the reference lines selection criteria. Thus, the extracted lines from the moving objects would not share the estimation of the transformation parameters. The mean execution time and number of iterations for 9 ms and 13.5, respectively.

### 4.3. Threshold Dependence

The proposed algorithm depends on a group of thresholds. The values of the thresholds are computed according to sensor precision and/or dynamics of the vehicle as described above. However, the proposed algorithm can achieve the exploration mission, even if the values of the thresholds are altered around the right computed values of the thresholds. Nevertheless, the altered threshold values depend on the environment of the exploration mission, and they will affect the sharing percentage between the two methods (RKF and ICP) in the entire mission.

**Figure 46:**
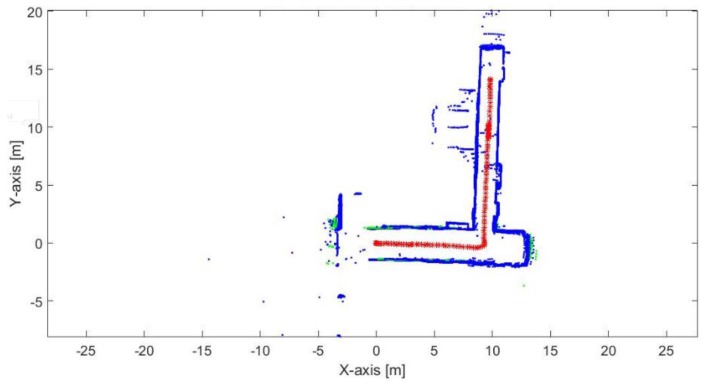
Mapping and position results for the proposed algorithm after changing values of the thresholds.

Figure 46 shows the mapping and position results for the proposed algorithm of the dataset V after changing some values of the thresholds. It is clear that the environmental structure was not affected by changing the values of the thresholds. The sharing percentage of the ICP algorithm for the entire dataset is 5.4% while the sharing percentage before changing the values of the thresholds is 1.5%. The mean execution time is 8.1 ms.

## 5. Conclusions

In this paper, a low-cost novel real-time scan matching algorithm, inspired by the video streaming broadcast key frame technique, is proposed. The proposed algorithm depends on a sole laser scanner range finder and does not need external aided sensors. The proposed algorithm endeavors to mitigate the accumulated errors that exist in the local and global scan matching, as the transformation matrix of the proposed algorithm is computed with respect to the previous key frame and not with respect to the previous scan. Initially, the proposed algorithm depends on selecting two reference lines from the extracted lines which compose the first reference key frame. The transformation parameters of all the consecutive frames are computed with respect to this first reference key frame, until new reference key frame is chosen. Thus, two new reference lines are swapped, and furthermore, the transformation parameters between the old and new reference lines are estimated. The transformation parameters of the next frames are computed with respect to the first reference key frame but taking into consideration the transformation parameters of the swapping process. For validating and evaluating the proposed algorithm, the mapping performance and time consumption are studied under different algorithms such as Hector SLAM, ICP, and feature to feature registration such as corners, in static and dynamic environments. It was found that the time consumption of the proposed algorithm is approximately reduced by 99%, 35.7%, 10% comparable with multi-level Hector SLAM, ICP, feature registration, respectively. The computational time of estimating the transformation parameters between each two successive scans is approximately 9 ms, which indicates the qualification of the proposed algorithm for real-time system implementations. 

Although the proposed algorithm depends on the availability of two non-parallel lines, it succeeds to provide a solution in the corridors and unstructured environment by switching to the ICP algorithm. The proposed algorithm is appropriate for dynamic environments, as the moving object features will not satisfy the reference lines selection criteria. Therefore, the extracted lines from the moving objects never compose a reference key frame.

## Figures and Tables

**Table 1:** Standard deviations of the laser scanner range finder according to the detection range.

Detection Range [m]	Standard Deviation (σ) [cm]
Less than 1	0.34
Less than 2	0.73
Less than 3	1.79
Less than 4	3.27
Less than 5	3.92
Less than 6	5.44

**Table 2:** Line availability and extraction time for different data sets.

Dataset Name	MIT Killian	MIT CSAIL	Intel Lab	ACES Building	Freiburg Building
Number of scans	17,481	1989	13,632	7375	4496
Mean number of lines	4.24	8.80	4.10	4.21	8.86
Percentage of more than three lines	88.2%	99.9%	81.3%	84.8%	99.8%
Mean execution time [ms]	3.7	8.9	3.4	4.0	7.4

**Table 3:** Computed parameters of the extracted lines of the first key frame.

Line Number	Coefficient of Determination $\left(R^{2}\right)$	Adjusted Coefficient of Determination $\left({\bar{R}}^{2}\right)$	Number of Data Points	Line Length [m]
1	0.9940	0.9937	51	5.7
2	0.9755	0.9706	13	1.0
3	0.8689	0.8631	48	5.4
4	0.9749	0.9722	22	2.8
5	0.9713	0.9598	8	1.8

**Table 4:** The environment status and contribution for each experimental dataset.

Dataset Name	Environment Status	Contribution
Dataset I	Static	Corridors, loopback, glass objects
Dataset II	Static	Brick walls, glass objects, Corridors
Dataset III	Static	Aluminum curtains, Sharp rotation (180°)
Dataset IV	Static	Glass objects, corridors
Dataset V	Dynamic	Glass objects, corridors, moving objects

**Table 5:** Mean execution time and number of iterations for multi-resolution map representation with different levels and grid cell dimensions.

Level	Cell Dimension [cm]	Mean Execution Time [s]	Mean Iterations Number
1	20	0.11	15.7
2	10, 5	0.80	33.5
3	20, 10, 5	1.15	47
